# The Expectation and Reality of the HepG2 Core Metabolic Profile

**DOI:** 10.3390/metabo13080908

**Published:** 2023-08-03

**Authors:** Olga I. Kiseleva, Ilya Y. Kurbatov, Viktoriia A. Arzumanian, Ekaterina V. Ilgisonis, Svyatoslav V. Zakharov, Ekaterina V. Poverennaya

**Affiliations:** 1 Institute of Biomedical Chemistry, Pogodinskaya Street, 10, 119121 Moscow, Russiailgisonis.ev@gmail.com (E.V.I.); k.poverennaya@gmail.com (E.V.P.); 2 Chemistry Department, Lomonosov Moscow State University, Leninskie gory Street, 1/3, 119991 Moscow, Russia; sviatoslav.zakharov@chemistry.msu.ru

**Keywords:** panoramic metabolomics, LC-MS, GC-MS, NMR, metabolic profile, HepG2, meta-analysis, data availability, data submission guidelines

## Abstract

To represent the composition of small molecules circulating in HepG2 cells and the formation of the “core” of characteristic metabolites that often attract researchers’ attention, we conducted a meta-analysis of 56 datasets obtained through metabolomic profiling via mass spectrometry and NMR. We highlighted the 288 most commonly studied compounds of diverse chemical nature and analyzed metabolic processes involving these small molecules. Building a complete map of the metabolome of a cell, which encompasses the diversity of possible impacts on it, is a severe challenge for the scientific community, which is faced not only with natural limitations of experimental technologies, but also with the absence of transparent and widely accepted standards for processing and presenting the obtained metabolomic data. Formulating our research design, we aimed to reveal metabolites crucial to the Hepg2 cell line, regardless of all chemical and/or physical impact factors. Unfortunately, the existing paradigm of data policy leads to a streetlight effect. When analyzing and reporting only target metabolites of interest, the community ignores the changes in the metabolomic landscape that hide many molecular secrets.

## 1. Introduction

Cancer cells’ metabolism prioritizes growth and biomass production, which can result in nutrient shortages and metabolic waste buildup occurring due to a lack of resources in the tissues around the cells. Cancer cells develop an assortment of metabolic adaptations to support their growth, which is influenced by the mutations possessed and the cell of origin. Some metabolites regulate gene expression, affect neighboring non-cancerous cells, and contribute to biomass and energy production [1,2,3].

HepG2 cells are commonly used as a model for studying liver cancer and drug metabolism due to their ability to maintain several liver-specific functions [4]. Having a high rate of proliferation and being easily cultured in vitro, HepG2 culture is a valuable tool used to study cancer machinery and nutrition patterns, as well as in drug discovery and toxicological studies. Establishing this core metabolome is crucial to gain insight into the cell’s vital molecular processes and practical purposes, such as drug discovery, toxicology research, and disease treatment. 

In biofluids and tissues metabolites vary significantly in concentration and chemical composition based on environmental conditions, exposure to drugs or toxic agents, and biological factors, making it impossible for a single approach to measure all of them at once [5,6,7]. Therefore, scientists use a combination of spectrometric techniques to obtain broad coverage of the metabolic space [8]. Metabolic profiling is typically performed using nuclear magnetic resonance (NMR) or mass spectrometry (MS) in tandem with gas (GC) or liquid (LC) chromatography. 

NMR does not require chemical manipulation of a sample, though it often suffers from poor sensitivity and difficulties in deconvolution and normalization of complex spectra, which often limits it to approximately 100 of the most abundant metabolites in a sample (for example, blood [9,10]). The field of metabolomics has witnessed notable instances of the utilization of this technique to achieve thorough characterization of the metabolome across various biological samples. For instance, a study conducted on human urine successfully identified and quantified a total of 209 metabolites [11]. These exceptional findings imply that the potential for metabolomic yield in cell culture or cell extract is likely to surpass that observed in blood or urine. 

MS often requires the derivatization of metabolites (especially when coupled with GC), though it provides the greater sensitivity required to measure a broader array of low-molecular-weight metabolites [12,13]. It is also be worth mentioning that the typical chromatographic conditions for LC-MS often favor lipids, whereas NMR is more focused on hydrophilic metabolites due to the aqueous solvents used in the analysis.

The metabolome constantly changes due to the fact that all chemical reactions occur in the cell. These changes result from the complex and non-linear interactions with metabolic, signaling, and regulatory pathways [14]. Each metabolomic experiment provides a “snapshot” of substances detected in analyzed organs and tissues [15]. 

Our study aimed to collect all existing snapshots of the molecular passport of the HepG2 cell line to evaluate the composition of the core metabolome of the cell line, which plays several essential roles in vital cellular processes. Additionally, we examined the composition of metabolites resulting from exposure to diverse chemical and physical agents that activate non-standard metabolic pathways. 

We present the core of the HepG2 metabolome as a set of metabolites systematically detected via MS or NMR across multiple experiments.

## 2. Materials and Methods

### 2.1. Source of the Data

To summarize recent research in the field of the HepG2 metabolome, we conducted a literature search by querying “HepG2 metabolome” in PubMed, which returned 313 articles (published during the period extending from 2006 to 2023) sorted based on relatedness to the query. A team of experienced metabolomics researchers followed a series of steps to retrieve study information from the literature, which involved reading bibliographic information and, in most cases, the full text of articles. 

We reviewed the list of suggested publications and selected a shortlist of the most suitable papers (published during the period extending from 2010 to 2023). The criteria we used to select articles were as follows:

1. The panoramic nature of the study (which supposes an intended comprehensive analysis of all of the detectable analytes in a biological sample, including chemical unknowns);
2. The access to results summarizing metabolomic findings.

In total, we found 56 datasets related to the impact of various factors.

### 2.2. Processing of the Data

In order to compare metabolite lists from various publications, we created Supplementary Table S1, which summarizes the chemical names of all of the metabolites and the details of the experiment in which it was identified (in total, 23,086 metabolic entries were included, which characterized 15,161 unique metabolites). This table also includes information on the intervention applied to HepG2 cells (such as drugs, genetic modifications, or exposure to toxins) and the analytical method used. To maintain accuracy, each metabolite was automatically assigned an InchiKey code using PubChem API [16]. About 90.1% of metabolites were converted successfully. In case of ambiguous identification, we used the oldest identifier or manually browsed metabolites (9.9%) via a direct search in PubChem.

For taxonomic annotation, we utilized the web service ClassyFire in combination with ChemOnt (version ClassyFire Batch created by Fiehn Lab [17]), which uses InChiKey ID to automatically assign chemical compounds to a taxonomy including more than four thousand different categories [18]. The original ClassyFire resource also has an option for batch loading. However, it accepts other types of identifiers (such as InChi String) as inputs, which limits its utility in our research.

By default, the metabolite was assigned a “Subclass” (medium—5th out of 11—represents the depth level of the compound’s classification in the ClassyFire system). Assigning a subclass to 2212 compounds was impossible; thus, we moved up a step in the taxonomic hierarchy and assigned a “Class” or “Superclass”. One hundred and seventy-five compounds were not assigned, even with higher levels of classification hierarchy; therefore, these compounds were excluded from further consideration. 

Further, we performed Metabolite Set Enrichment Analysis via MetaboAnalyst v. 5.0 [19] to highlight any meaningful patterns that could be discerned in the groups of metabolites detected in HepG2 cells. Over-representation analysis was implemented using the hypergeometric test to evaluate whether a particular metabolite set was represented more than expected by chance within the dataset.

### 2.3. Limits of Our Approach

Each paper’s lists of identified metabolites were manually extracted from the articles, supplementary data, and data repositories. The majority of the metabolites were identified based on their chemical names. These names can be expressed in various ways, including systematic names (such as “propane-1,3-diol”), semi-systematic names (like “diacetylmorphine”), non-systematic—trivial—names (such as “tartaric acid”), abbreviations (like “UTP”), formulas (such as “C27H46O”), names of fragments (like “steroid derivative”), families of compounds (such as “fatty acids”), and adjective forms (like “taurocholic”). Most of the names mentioned above do not provide precise details regarding the structure of the compound (such as the connections between atoms and bonds), making them unsuitable for fulfilling the demands of the scientists who retrieve information on structural characteristics [20].

In order to unify the obtained data, we converted all of the chemical names to Inchi keys identifiers, which represent hashed information on the structures of the compounds. Near 1% of chemical names were left unidentified because chemical names provided in publications were incorrect or misleading in the sense that they contained mistakes that made it impossible to generate a structure based on the name as it was published by authors of accumulated articles [20]). 

Among the main problems, hindering the flow and reanalysis of metabolomic data, grammar mistakes (e.g., billirubin instead of bilirubin), misprints (pvruvate instead of pyruvate), and syntax (parentheses, apostrophes, missing or extra spaces, e.g., PE(18:2(9Z12Z)20:5(5Z8Z11Z14Z17Z)) instead of PE(18:2(9Z,12Z)20:5(5Z,8Z,11Z,14Z,17Z))) were most prevalent.

## 3. Results and Discussion

We investigated the meta-metabolome of HepG2 cells and the general trends in conducting metabolomic studies and presented the results obtained. In our study, we summarized the results of 56 projects performed using LC-MS (33 articles), GC-MS (16 articles), and NMR (14 articles). We also looked at the results of four research projects performed using less common technologies, in particular CE-MS [21], IM-MS [22], direct MS-MS [23], and SPI-TOF-MS [24]. It is noteworthy that our meta-analysis exclusively relied on the outcomes of metabolomic analysis as reported in chosen publications, without performing any additional reprocessing.

Immortalized hepatocyte culture is one of the most studied closed biosystems from a metabolomic point of view: it is located in the top three regarding the number of metabolomic publications in PubMed, along with HEK293 and MCF-7 cells. Next, we presented a complete list of small molecules registered in the HepG2 cell line, information on the most frequently detected metabolites and their involvement in metabolic pathways, features of the metabolomic profiles of the object under study, and “travel essays” on data deposition and re-analysis that we formed during the study.

### 3.1. Impact on HepG2 Cell Culture: “Control” and “Experimental” Datasets

**Figure 1:**
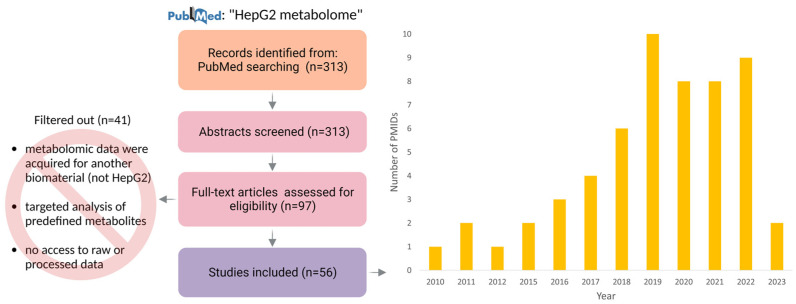
Formation of publication trends among articles that present results of HepG2 cell metabolomic profiling: distribution of thematic publications by year according to the PubMed database. The corpus of 56 papers that were presented in the study was derived through a review of the full-text versions of 97 articles. The exclusion of certain articles from consideration was primarily attributed to the absence of access to either raw or processed metabolomic data, the study’s targeted nature, or the execution of metabolomic experiments using a biomaterial type other than HepG2.

In Figure 1, we present a diagram of the PubMed search process for collecting and evaluating articles to establish a representative pool of studies that investigated the metabolome of HepG2 cells. The cumulative dataset that we used for the meta-analysis of reported metabolites in HepG2 cell culture consisted of 56 articles (Supplementary Table S2). These articles were published in reputable peer-reviewed journals over a period spanning 13 years, i.e., from 2010 to 2023, as illustrated in Figure 1. The majority of the journals included in this dataset were categorized within the Q1 and Q2 quartiles, as shown in Supplementary Figure S1. Such journals are widely acknowledged for their high impact factors, with Q1 representing the top 25% of journals and Q2 encompassing the following 26–50% range.

Despite having similar genetic backgrounds, different examples of HepG2 cells (genetically modified or treated with various drugs and toxic agents) can display vastly different metabolic phenotypes [25,26,27]. Utilizing this variability could provide insights into the abnormal tumor metabolism and machinery of hepatocytes. 

Nine datasets described situations in which cells were not subjected to explicit chemical or physical stimuli [21,22,27,28,29,30,31,32,33]. The article by Chun-Yun Zhang et al. [31] is particularly interesting. It investigated the characteristics of the conversion of 3,3′-dichlorobiphenyl (PCB11) in HepG2 cells, which were exposed to PCB11 in DMSO for 24 h, and the effect on cell metabolome was monitored in comparison to the DMSO-control. The metabolome of the HepG2 culture exposed to the neurotoxin PCB11 was evaluated using non-targeted high-resolution LC-MS. As is often the case, since the article’s focus was on evaluating the impact of PCB11, the panoramic metabolome of the control sample was not published, but it was kindly provided by the author upon request via email and used in this meta-analysis as one of the “no impact” datasets. Careful annotation of experimental data (which primarily occurred if some data did not directly support conclusions made in the manuscript) is undoubtedly burdensome; thus, “redundant” data were not made publicly available [34].

In the vast majority of articles, the motivation of researchers was to assess the perturbations of the metabolome of the chosen culture in response to various stimuli. The investigated impacts naturally had a wide range. 

The effect of using drugs of various natures was evaluated in 15 publications, [24,35,36,37,38,39,40,41,42,43,44,45,46,47,48]. Four publications studied the disturbance of the HepG2 metabolome after genetic editing [49,50,51,52]. Moreover, the impact of a combination of drug use and genetic technologies was established in two scientific articles [53,54]. 

The significance of the HepG2 line as a model for toxicological research is difficult to overestimate—the line is routinely used to determine the toxicological profile of different substances and predict the toxicity of new compounds in a highly controllable mode. In our collected data pool, 10 articles described the influence of various toxic agents on the HepG2 metabolome [25,55,56,57,58,59,60,61,62].

Seven papers of accumulated datasets studied the effects of sugars [63,64], acids [65,66], and other “neutral” compounds [67,68,69]. One work was devoted to the impact of gamma-irradiation [70] on the HepG2 cell line.

The remaining eight articles evaluated the effect of small-sized particles (nanomaterials—six articles [23,71,72,73,74,75] and technogenic ultrafine particles—two articles [76,77]) on the cell culture.

Out of 56 articles, 4 had a direct link to raw data (from which one valuable dataset was kindly provided by the authors upon e-mail request [31], and three others deposited their data in a repository [27,46,68]). Twenty-three articles provided processed data in a reduced form (i.e., as complete lists of detected metabolites) [21,22,25,28,29,30,32,33,35,36,39,43,50,55,57,58,63,64,70,72,73,74,78]. The remaining 29 articles [23,24,37,38,40,41,42,44,45,47,48,49,51,52,53,54,56,59,60,61,62,65,66,67,69,71,75,76,77] directly demonstrated fragmentary information about metabolites in the manuscript or Supplementary Materials, such as by providing tables, heat maps, or schemes that only noted changing metabolites (Supplementary Table S2).

### 3.2. The Resemblance of an Average Dataset in Accordance with the Number of Reported Metabolites 

**Figure 2:**
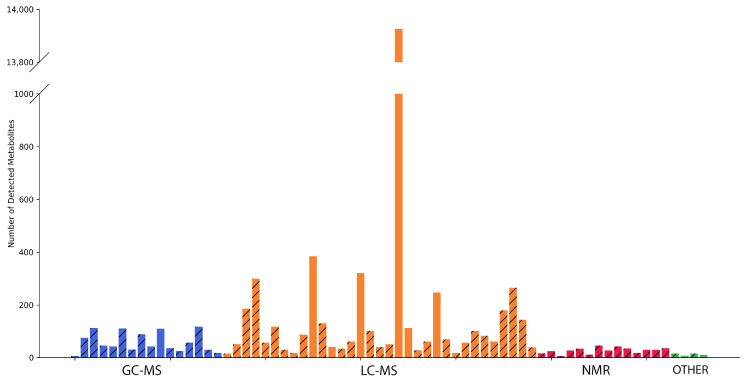
A histogram of the distribution of the number of HepG2 metabolites per article. Articles are grouped based on the analytical methods (GC-MS, LC-MS, NMR, and others) used. The hatched columns belong to experiments where HepG2 cells were treated. The columns filled with colors reflect control—“no impact”—experiments.

On average, one experiment reported about 331 findings (with a maximum of 13,926 unique metabolites, a minimum of seven metabolites, and median of 46 metabolites; Figure 2). Obviously, the number of findings was not balanced. There was a clear leader, i.e., [31], which reported a record number of findings, and several unexpectedly small datasets, i.e., [21,23,71]. Excluding the data derived from [31], the average number of metabolites provided in the article was 84, with a maximum of 385, a minimum of 7, and a median of 46.

The manuscript examining the complex mechanism of toxicity of multi-walled carbon nanotubes presented 11 different metabolites detected via NMR [71]. The fact that the NMR-generated data array is expectedly much narrower than the list of metabolites detected via chromato-mass spectrometric methods (on average, about 27 metabolites for NMR versus 665 for LC-MS and 60 for GC-MS) was aggravated by the decision of the authors to present only variable metabolites. From this perspective, the “metabolome” presented may seem modest and, perhaps, does not fully demonstrate the full analytical power of the NMR technology. As such, NMR may be limited in its ability to detect low-abundance metabolites and may only identify a relatively small number of metabolites in complex mixtures (ranging from 20 to 200 unique substances, depending on the resolution of NMR) in comparison to MS (which can potentially identify over 1000 substances). This advantage makes mass spectrometry a dominant choice for exploring a wide range of metabolites. However, due to its ease of sample preparation and exceptional reproducibility, NMR has proven to be a reliable method for metabolomic profiling, but it is possibly not the best method for identifying unknown compounds from a complex solution.

Against the background of NMR-based articles, the varied natures of MS datasets are noteworthy, making up 98% of the collected experimental array. However, the popularity and performance of this platform do not guarantee the completeness of the published data. Despite the panoramic nature of the works collected via our meta-analysis, in [75], which investigated the effect of strigol/albumin/chitosan (S/A/CNP) nanoparticles on the HepG2 cell line, only 18 metabolites were published. This subset is not exhaustive and likely represents only a fraction of the full complement of metabolites affected by nanoparticle exposure. Specifically, the 18 metabolites identified in this study exhibited log2FC values ranging from −3.45 to 2.10, indicating a range of fold changes in abundance. The presented data are much more modest than expected when using advanced chromato–mass–spectrometric technologies and do not allow their reanalysis to be carried out effectively by other scientists. Regrettably, the lack of open data associated with this study precludes the possibility of the independent verification or reproducibility of these findings.

### 3.3. The Potential of Analytical Methods to Detect Different Chemical Substances

The physicochemical properties of the target metabolites determine the choice of analytical technology [15]. We analyzed the list of subclasses of chemical compounds detected via various methods (intersections and unique metabolite subclasses and the complete list of subclasses are given in Table S3). The result illustrated the well-known strength of the LC-MS method, which provides the maximum width of the spectrum of physicochemical properties of the detected compounds. The tandem of LC and MS made it possible to identify 609 subclasses of metabolites, and most of them (507 subclasses mapped to 200 classes) could not be detected via GC-MS, NMR, and other MS-based technologies. Thus, not only exotic compounds (e.g., isocoumarams and cinchona alkaloids), but also rather basic pyrrolizines, naphthalenes, and pyrans, were included among the “unique” metabolites determined via LC-MS.

The second place in terms of coverage is occupied by GC-MS, which made it possible to detect a total of 93 subclasses, and five of them, which were related to homogeneous non-metal compounds and miscellaneous mixed metal/non-metals, were not available for other technologies.

The NMR method allowed the detection of 39 subclasses of metabolites, only 1 of which (homogeneous actinide compound) was exclusively detected using this technology.

**Figure 3:**
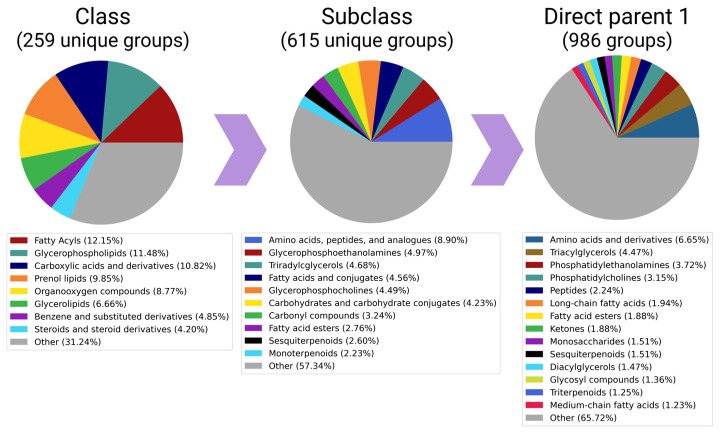
Detectability of metabolites of different chemical natures: pie-charts presenting proportions of the classes, subclasses, and direct parents among all (15,161) metabolites, which are presented in a cumulative pool of 56 articles. Subclasses that accounted for less than 2% (or 1% in case of “Classes” and “Direct parents 1”) of unique metabolites were merged into the “other” categories.

The total “core” of subclasses of metabolites was made up of the most studied metabolites, namely glycerophosphocholines (involved in cell membrane synthesis and degradation), purine and pyrimidine ribonucleotides (major energy carriers), glycerophosphoethanolamines (involved in the secretion of lipoproteins in the liver), and amino acids and peptides (building materials for protein synthesis). This pattern is illustrated by the pie chart (Figure 3), which reflects the proportion of the subclass among all detected metabolites in the HepG2 line.

In order to analyze the data from different hierarchical levels, we utilized pie charts to visually represent “Classes” (a broader classification) and “Direct parents 1” (a more specific classification based on the compound’s largest structural feature). This approach allows a comprehensive understanding of the chemical composition of metabolites in the HepG2 cell line. However, it also highlights the limitations of the automatic grouping of metabolites using the ClassyFire system, which heavily relies on pre-defined chemical patterns. The pie chart for the “Subclass” category reveals that amino acids are the most frequently detected group. Although there are significantly fewer entries for “Carboxylic acids and derivatives” (the class to which amino acids belong) than lipids (which encompass glycerophospholipids, phenol lipids, and glycerolipids), amino acids and derivatives still hold the top position at the “direct parent” level.

### 3.4. Most Often “Published” Metabolites and their Involvement in Biological Processes

During our meta-analysis, it became apparent that it is not only families of metabolites (in terms of belonging to chemical subclasses) that are systematically detected, but also distinct metabolites.

We evaluated the occurrence of metabolites in published studies (Supplementary Table S4) based on LC-MS, GC-MS, and NMR technologies. We subdivided the entire pool of unique metabolites into those reported “more often without impact” (if the metabolite was more often described in articles in which the cell line was untreated), “more often under the impact” (if the metabolite was more often detected in articles in which HepG2 was subjected to chemical or physical treatment), and “general” (the most frequently described metabolites in the literature, regardless of context).

**Figure 4:**
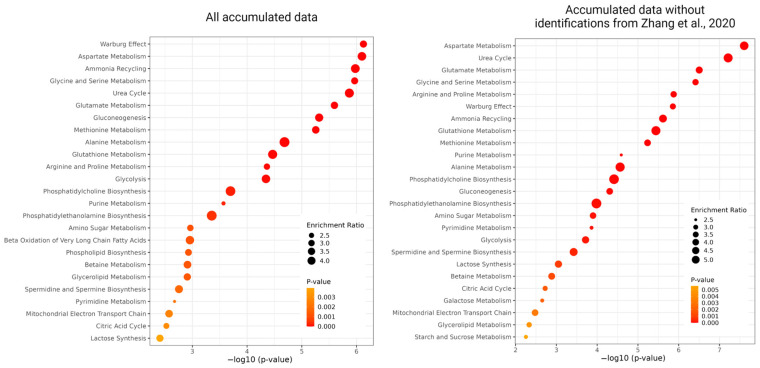
The results of the over-representation analysis of the most enriched metabolomics sets are as follows: the top-25 enriched sets built on metabolites from all accumulated metabolic data (left) and data excluding ref. [31].

As frequently detected, we selected 288 metabolites reported by the authors of more than five articles. Consequently, we found that the endo- and exo-metabolites that comprised this pool of 288 small molecules significantly overlapped, with only 37 metabolites exclusively detected in studies focusing on cells (Supplementary Table S5). Analysis of the sum of these metabolites using the MetaboAnalyst platform showed that such a dataset was enriched in the participants of several processes typical of a cancer cell line, which is primarily designed to facilitate the uptake and incorporation of nutrients into the biomass needed to produce a new cell. Among these typical processes are the Warburg effect, metabolism of various amino acids, ammonia recycling, gluconeogenesis and glycolysis, urea and citric acids cycles, phospholipid biosynthesis, and purine and pyrimidine metabolisms (Supplementary Table S4 and Figure 4). It is worth mentioning that the selection of the top-25 enriched sets for HepG2 metabolites remains largely consistent, even when excluding [31] from the overall dataset that comprised 56 articles. Among these pathways, 23 were consistently identified in both scenarios, indicating their robustness. The two pathways that differed between the two datasets were Galactose metabolism and starch and sucrose metabolisms, which were characteristic of the “reduced” dataset, while beta oxidation of very long chain fatty acids and phospholipid biosynthesis were specific to the complete dataset. 

Frequent and reliable detection of such metabolites gives hope that the participants in these processes can “mirror” the perturbations of the corresponding processes and become the basis of biomarker panels.

Given the above facts, comparing the processes to which the metabolites detected in treated and untreated cells are mapped was fascinating. We expected to see an intersection of 15 of the top-25 processes occurring in both cell types (purine and pyrimidine metabolisms, amino acids metabolism, phosphatidylethanolamine biosynthesis, and biosynthesis of polyamines involved in the regulation of genetic processes from DNA synthesis to cell migration, proliferation, differentiation, and apoptosis). Among untreated cells, the specific degradation and shuttle of several amino acids, phenylacetate metabolism, nicotinate, and nicotinamide metabolism have been noted. In addition to these processes, while maintaining cellular homeostasis, the synthesis of thyroid hormone is notable, which regulates downstream the expression of a large panel of genes that support cancer cell proliferation, antiapoptosis, and cancer-associated angiogenesis [79]. 

For treated HepG2 cells, a clear pattern is observed—most often, the metabolites detected are involved in the reorganization of metabolic pathways in cancer (e.g., Warburg effect, gluconeogenesis, glycolysis, and beta-oxidation of fatty acids). In response to exposure to various natures, the cancer cell line reacts with the above processes. Such core “alarm” tumor processes and the metabolites that participate in them should be explored in more detail as potential targets.

We believe that it is essential to assemble the HepG2 metabolomic core, no matter the meaning of this concept (which can be a subset of metabolites constantly identified in the cells due to the limitations of analytical techniques or a pool of small molecules essential for the basic functioning of a cell). Studying the core metabolome of the cancer cell line can help to reveal dysregulated metabolic pathways and unravel the underlying mechanisms driving tumor growth and survival. Understanding the core metabolome provides insights into potential therapeutic targets and the analytical capabilities of the applied methods.

### 3.5. Travel Essays on the Way to the Formation of the Metabolomic Core of the HepG2 Culture

Even though most metabolomics studies are conducted in full-scan mode, and the pre-experiment sample preparation does not close the window of opportunity for identifying a wide range of compounds, the manner in which results are presented is such that only a portion of the findings is demonstrated (only metabolites whose content is significantly altered under a particular investigated condition or treatment). This situation suggests a metabolomic “deja vu” effect in which the same metabolites are reported [80], predominating regardless of the experiment. As in proteomics, data-driven metabolomics faces the following question—do we permanently observe experimental artifacts or universal sensors that respond to any disturbance?

In order to answer this question, it is necessary to re-analyze a representative pool of published data, the protocols for the obtainment of which are transparent during the metabolomic experiment and further processing. In the general case, this idea is broken by the natural tendency of researchers to share results rather than data. “Raw” data are usually not presented, and the result of processing is often shown in fragments [81].

The deposition of initial data in open-access depositories in other omics sciences has long been a self-evident requirement for the publication of findings; however, in metabolomics, the rules are much less stringent. Many journals that publish metabolomic studies either do not have a publication policy regarding the provision of experimental data at all or do not require but only “encourage” open publication of data [81]; thus, scientists are expected to spare themselves the burdensome task of annotating and publishing complete experimental data. Even when data were published via thematic repositories, critical information about the experiment may be lost. For example, it has been observed that metadata concerning the operation of a mass spectrometer are usually (although not consistently) reported. However, the details of chromatographic separation are often (in 70–80% of cases) insufficiently described [82]. Accordingly, only circa 20% of the descriptions allow data to be reused (e.g., to predict the retention times of specific metabolites). 

We witness an inconsistency in using recommended reporting standards in many excellent manuscripts produced at a high scientific level and published in high-ranking journals [72]. Thus, among the studies that we have summarized, which were published over the past 13 years in respectable journals, only a few (for example, [33]) fully adhere to the system recommended by the Metabolomics Society for classifying identifiable metabolites based on levels of confidence [83], ranging from confidently identified to unknown. The authors of scientific publications are advised to provide transparency regarding the criteria used to ensure the validity of the data analyzed in their studies, as per standardization guidelines. Researchers are equipped with two options when interpreting a chromatogram. The first approach involves utilizing pure standards (and/or isotopically labeled standards) to determine retention times or indexes, followed by obtaining a characteristic mass spectrum that can be accurately matched with a library mass spectrum. This method enables precise interpretation of mass spectrometric data. In contrast, the second (more popular) option involves utilizing various libraries or databases to search for potential candidates in an operator-dependent manner, which poses a potential risk of causing inadequate analysis of the acquired data, as a real spectrum may contain numerous peaks that do not necessarily correspond to specific metabolites.

Attempts to reanalyze and comprehend already processed data are also hampered by the unstructured mechanism used to assign names to metabolites. There are many different formats and styles used to name molecules, including InChIKey, SMILES, PubChem, ChemSpider, CHEBI, and many others, in addition to traditional names, resulting in the redundancy of multiple (often conflicting) names of the same molecule.

Despite clear and well-established guidelines, MSI recommendations are followed by a minority of researchers, and only 7% of publications show identifications of the highest level of reliability [84]. Several reasons for this problem exist, including the increasing trend of research teams outsourcing LC-MS analysis to third-party entities (e.g., core facilities or private enterprises). These entities provide finalized reports, but they often do not disclose much information about their proprietary methods or in-house spectral libraries. The raw data may be kept by a third party and are often not shared with authors or deposited in metabolomics repositories. 

The example of a popular object clearly shows how voluminous, intricate, diverse, and uncertain data, compounded by inadequate archiving, create enormous challenges for reporting scientific research that adheres to the principles of FAIR (Findable, Accessible, Interoperable, and Reusable) science [85].

## 4. Conclusions

This manuscript describes HepG2 cells’ metabolome and its routinely reported fraction, as registered through MS techniques and nuclear magnetic resonance analysis. In our study, we collected information on 15,161 metabolites previously detected in HepG2 cells. Meta-analysis of published data showed that even in panoramic studies, scientists focus on specific metabolites, ignoring the rest of the metabolomic profile. Interestingly, these metabolites are repeated from study to study (in our case, 288 metabolites), which, on one hand, may indirectly confirm their key role in the metabolism of hepatocytes, and, on the other hand, indicate significant limitations of technologies that only allow high-reliability identification for these compounds. 

We demonstrated that the comprehensive list of identified metabolites is often not fully disclosed. In most cases, the focus of researchers primarily concerns specific metabolites of interest, disregarding other compounds. As a result, third-party researchers have to analyze only the “published “metabolome, rather than the complete set of detected metabolites. These “deja vu” metabolites are consistently reported across different studies, which, on one hand, may indirectly confirm their crucial role in cancer metabolism. On the other hand, this phenomenon may highlight significant technological limitations if identification is reliable only for these specific compounds. 

In addition to artificially narrowing the width of panoramic data, we encountered the phenomenon of data closure. Our research sheds light on the current state of metabolomics data. Despite the publication of MSI guidelines in 2007 [86] and the FAIR Data Principles in 2016 [87], the landscape of metabolomics data remains quite ambiguous, in contrast to proteomics [88], where data reanalysis and meta-analysis serve as powerful tools for data verification and tracking technology and software advancements. 

Despite advancements in generating high-resolution spectral profiles, interpretation of metabolomic data still largely relies on expert intuition and remains a significant challenge [89,90,91]. We acknowledge that there is still uncertainty regarding the assessment of retention indices (as well as quality criteria required for high-resolution MS) in the published data. 

Data closure is accompanied not only by the complexity of the process itself, but also by the policies of thematic journals, which often do not require authors to place experimental data in public repositories, making it impossible to verify data and make meaningful further use of them [81]. Only 5% of studies have made their raw data publicly available. The same problem applies to metadata, as detailed protocols are only found in a limited number of studies and are not accessible through public repositories, rather existing within the texts of articles or their Supplementary Materials. The unavailability of raw data reduces the opportunity to verify and meaningfully re-use them [92,93].

The limited throughput of classical techniques, the lack of precise presentation rules available to compare metabolic profiles, and “one-time data” are the main bottlenecks encountered on the way to forming the molecular passport of a biological object. However, we have made efforts to provide a comprehensive analysis by including studies that followed MSI guidelines and independently evaluating the validity of metabolite identifications in other articles. Being fully aware of the difficulty and pain involved in the process of metabolite identification and further depositing experimental data in open repositories, we would like to emphasize how important it is for the entire metabolomics community to extract value and meaning from the time and resources expended when performing untargeted analyses. 

## Supplementary Materials

The following supporting information can be downloaded via the below link: https://www.mdpi.com/article/10.3390/metabo13080908/s1, Figure S1: Information on the ranking and popularity of scientific journals preferred by authors for publishing metabolomic studies; Table S1: Cumulative data on metabolites detected in HepG2 cell line; Table S2: Map of various types of chemical or physical impacts on HepG2 cell line presented in scientific literature; Table S3: Contents of Venn diagram presenting subclasses of metabolites detected via various analytical technologies; Table S4: Metabolite frequencies in publications describing various experiments (three analytical methods and the absence or presence of influence on the HepG2 cell line are taken into account); Table S5: Distribution of 288 often-published metabolites between endo- and exo-metabolomes.
