# Supplementary figures and images for: The Expectation and Reality of the HepG2 Core Metabolic Profile
Source: Metabolites. 2023 Aug 3;13(8):908. doi: 10.3390/metabo13080908 (PMC10456947; doi:10.3390/metabo13080908)

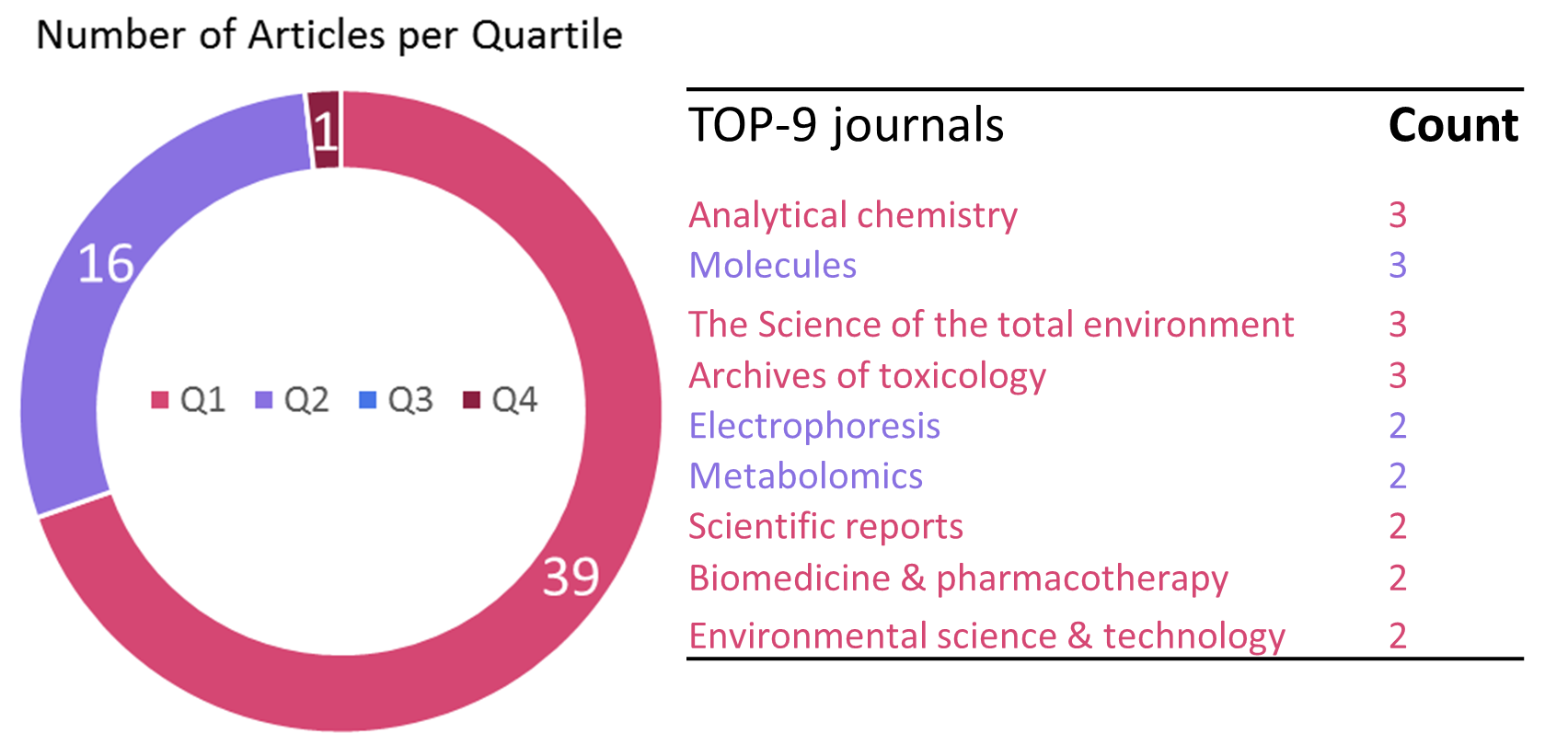

Supplement: Supplementary file 1 [file metabolites-13-00908-s001.zip › metabolites-2442665-supplementary (2)/supplementary/suppl_fig_1.png]
